# A Histone Deacetylase Activity Model for the Discovery and Validation of Sepsis Biomarkers

**DOI:** 10.2174/0118715303458056260211101424

**Published:** 2026-04-08

**Authors:** Yuanyuan Wang, Tao Shu, Yanpeng Li, Qiangqiang Kang, Zhansheng Jia, Chunyan Ma

**Affiliations:** 1 Department of Infectious Diseases and Hepatology, Xi'an International Medical Center Hospital, Xi'an, 710100, China;; 2 Department of Urology, Xi'an International Medical Center Hospital, Xi'an, 710100, China

**Keywords:** Sepsis, histone deacetylase, biomarker, immune infiltration, machine learning, molecular docking

## Abstract

**Introduction:**

Histone deacetylases (HDACs) play critical roles in immune regulation and inflammatory responses in sepsis. This study identified HDAC-related genes and explored their potential roles in sepsis.

**Materials and Methods:**

HDAC scores were computed by ssGSEA. WGCNA was applied to identify HDAC-related gene modules, followed by functional enrichment analysis. Machine learning methods were employed to screen diagnostic biomarkers. Immune infiltration analysis and molecular docking were performed to validate gene-immune correlations and potential drugs. An *in-vitro* sepsis model was established by using lipopolysaccharide to induce THP-1-derived macrophages. The gene expression and inflammatory factor levels were assessed using qPCR, Western blot, and ELISA methods. Finally, the effect of *TRIM24* knockdown on cell apoptosis and inflammatory responses was analyzed.

**Results:**

Two HDAC-related genes (*TOM1L2*, *TRIM24*) were identified as potential diagnostic biomarkers for sepsis, showing significant correlation with immune cell infiltration. Molecular docking confirmed the binding capacity of Acetaminophen, 8-azaguanine, and Ochratoxin A with the two biomarkers. *In-vitro* sepsis model showed that *TRIM24* knockdown markedly lowered the levels of pro-inflammatory cytokines and apoptosis.

**Discussion:**

The two biomarkers, along with the RiskScore model, could potentially contribute to the prognostic assessment of sepsis and the development of personalized treatment. However, further experimental validation is also needed to substantiate our findings and to translate them into practical clinical applications.

**Conclusion:**

This study identified *TOM1L2* and *TRIM24* as key HDAC-related biomarkers for sepsis, providing novel insights for the early diagnosis and targeted treatments of sepsis.

## 1 INTRODUCTION

Sepsis is characterized by organ dysfunction due to dysregulated host response to infection, with septic shock being its most severe form [1, 2]. Global data reported 48.9 million cases of sepsis worldwide, with a death rate of 22.5%, accounting for around 20% of total global mortality [3, 4]. Over the past few decades, though multiple methods have significantly lowered the mortality of sepsis, the overall mortality still remains high, and the therapeutic effect still needs to be improved [5]. At present, early diagnosis and intervention of sepsis in clinical practice face significant challenges due to the lack of sensitive and specific biomarkers.

Epigenetic mechanisms mediate heritable phenotypic changes without altering the DNA sequence, and disruption of epigenetics-related gene expression can cause cancers, autoimmune diseases, and some other diseases [6, 7]. Recently, the role of epigenetics in the pathogenesis of sepsis has gained increasing attention. In particular, histone acetylation is a key epigenetic modification implicated in multiple biological processes, including inflammatory response, immune regulation, and cell metabolism. Alterations in histone acetylation are closely associated with immune dysfunction and may play an important role in the development of sepsis [8, 9]. Specifically, by regulating the expressions of key genes associated with inflammation, immunity, and cell survival, histone acetylation is involved in the pathological process of sepsis, including host response dysregulation and organ dysfunction. Therefore, targeting histone deacetylases (HDACs) and histone acetyltransferases (HATs) might be a promising new therapeutic direction [8].

This study aimed to uncover novel and functionally relevant biomarkers for sepsis by leveraging the dysregulated epigenetic landscape, specifically the HDAC activity model, as a strategic discovery framework. Its primary innovation lay in the integrative approach that moved beyond simple differential expression analysis. Rather than commencing with conventional differential expression analysis, this study anchored its inquiry in the coherent pathological context of global HDAC-associated dysregulation. We employed a multi-stage computational pipeline coupled with experimental validation to ensure the biological and clinical relevance of the findings. The research logic proceeded from establishing an HDAC-associated context, to identifying co-expression gene modules within it, screening for robust diagnostic candidates, and ultimately focusing on functional characterization of a top-priority candidate in a disease-relevant cellular model. The identified biomarkers could contribute to the early detection of sepsis, and the functional elucidation of a key gene provided a direct experimental basis for developing targeted therapeutic strategies aimed at modulating the inflammatory and immune responses central to sepsis pathology.

## 2 MATERIALS AND METHODS

### 2.1 Data Acquisition

The chip data of GSE89376 and GSE57065 were downloaded from GEO [10]. The probes were transformed into Symbols based on the annotation files. Samples without clinical follow-up data were excluded. A total of 12 normal samples and 12 sepsis samples were obtained from GSE89376 (the training set), while 25 normal samples and 82 sepsis samples from GSE57065 (the independent validation set) were retained.

A total of 73 HDACs were obtained from a previous study [11]. This curated gene set, which encompassed major members across different HDAC classes, was adopted to ensure a comprehensive representation of HDAC-related transcriptional activity for subsequent signature scoring.

### 2.2 The Relationship Between HDACs and Immunity

The scores of HDAC-related genes were calculated by ssGSEA. The infiltration of 10 immune cell types was assessed using MCP-Counter package [12]. The association between HDAC signature scores and infiltration of various immune cells was further analyzed.

### 2.3 Weighted Gene Co-Expression Network Analysis (WGCNA)

WGCNA [13] was employed to delineate co-expression gene modules. After clustering samples to ensure data quality, we determined the appropriate soft threshold (β) utilizing the pickSoftThreshold function to approximate a scale-free network. Subsequently, we built a Topological Overlap Matrix (TOM) from the expression data and performed hierarchical clustering to cluster genes. Modules were clustered *via* the dynamic tree cut method and then merged based on eigengene similarity, with the parameters set as height=0.25, deepSplit=2, and minModuleSize=100 [14].

### 2.4 Enrichment Analysis

To explore the biological functions of genes within the selected co-expression modules, Gene Ontology (GO) enrichment analysis [15] was performed using the DAVID database [16].

### 2.5 Machine Learning

LASSO regression analysis was implemented *via* the R package glmnet [17], with 10-fold cross-validation (nfolds=10 and family=‘binomial’) to select feature factors.

Employing the R package randomForest [18], model performance was optimized by tuning the parameters mtry and ntree, with the Out-of-Bag (OOB) error serving as the evaluation metric. Gene importance was evaluated according to Mean Decrease in Accuracy (MDA) and the Mean Decrease in Gini index (MDG). The top 10 important genes were selected for further analysis.

### 2.6 Immune Infiltration Analysis and Gene Enrichment Analysis

The ssGSEA function of GSVA package [19] was employed to calculate the scores of 28 types of Tumor-Infiltrating Immune Cells (TIICs). The MCP-counter package [20] was employed to compute the scores of 10 types of immune cells. The ESTIMATE package [21] was used to calculate the immune score.

### 2.7 Targeted Drug Prediction and Molecular Docking

The screened hub genes were subjected to enrichment analysis using the Enrichr package [22] and drug-target prediction using the DSigDB database. The crystal structures of receptor proteins were obtained from the UniProt database (https://www.uniprot.org/), prioritizing structures with an X-ray source, lower Å value, longer amino acid sequences, and single-chain composition. Based on the drug prediction results, the 3D structures of the targeted drugs were downloaded from the PubChem website (https://pubchem.ncbi.nlm.nih.gov/) [23] as ligands. For molecules lacking 3D structures or requiring gene modulation, alternatives were selected based on the *P*-value. All molecules were processed using PyMOL software [24] to remove water molecules and small molecules and add hydrogen atoms. The 3D structures of the targeted drugs were energy-minimized using ChemBioOffice software. Subsequently, molecular docking between the receptor protein and drug molecules was performed using AutoDockTools under the screening criteria of binding energy < -5 kcal/mol and hydrogen bond<3.5 Å.

### 2.8 Cell Culture and Sepsis Model Establishment

Human monocytes (THP-1, TIB-202, ATCC, Manassas, Virgina, USA) were commercially obtained. THP-1 cells were cultivated in RPMI-1640 medium (30-2001, ATCC, USA) filled with 10% FBS and 100 U/mL penicillin at 37°C in an incubator in 5% CO_2_.

To establish an *in vitro* sepsis model, THP-1 monocytes were treated with 100 nM phorbol 12-myristate 13-acetate (PMA) (S1819, Beyotime, China) for 48 hours to differentiate into macrophages, referred to as THP-1-derived macrophages. Post differentiation, these macrophages were stimulated with 1 µg/mL LPS (L2880, Sigma-Aldrich, Germany) for 24 hours to induce an inflammatory response akin to sepsis.

### 2.9 Cell Transfection

The siRNAs targeting *TRIM24* (si-*TRIM24*#1: 5'-TACAGTAGAAAAGTCAAATCAGG-3'; si-*TRIM24*#2: 5'-AAGTCAAATCAGGTATGTACAAG -3') and si-NC were provided by Shanghai GenePharma Co., Ltd. for the downregulation of *TRIM24* expression in THP-1-derived macrophages. The transfection of si-*TRIM24* into THP-1-derived macrophages was carried out using Lipofectamine 2000 transfection reagent kit (11668027, Invitrogen, Carlsbad, California, USA).

### 2.10 RNA Extraction and qRT-PCR

Firstly, total RNA was extracted from LPS-treated or untreated THP-1-derived macrophages using the TriZol RNA extraction kit (15596026CN, Invitrogen, Carlsbad, California, USA). After assessing RNA concentration and purity, cDNA was synthesized with the SuperRT cDNA synthesis kit (S665-100T, Takara Bio, Shanghai, China). Following this, qRT-PCR was performed using the SYBR Green qPCR mix (D7260, Vazyme, Shanghai, China). Finally, data analysis was conducted *via* the 2^-ΔΔCT^ method, with *GAPDH* as an endogenous reference gene. The specific primer sequences were presented in Table **1**.

### 2.11 Immunoblotting Assay

Following treatment, cells were collected and lysed using RIPA lysis buffer (P0013B, Beyotime, Shanghai, China). Protein concentrations were determined by the BCA protein assay kit (P0009, Beyotime, Shanghai, China) to ensure equal loading. Equal amounts of protein were equally separated by SDS-PAGE gel (P0012A, Beyotime, Shanghai, China) and transferred onto PVDF films (FFP33, Beyotime, Shanghai, China). After blocking the films with TBST buffer containing 5% non-fat milk (ST665, Beyotime, Shanghai, China) for at least 1 hour, primary antibodies against *TOM1L2* (ab121716, 1:1000, Abcam), *TRIM24* (ab174287, 1:1000, Abcam), cleaved caspase 3 (ab2302, 1:500, Abcam), BAX (ab32503, 1:1000, Abcam), BCL2 (ab182858, 1:2000, Abcam), and internal reference β-actin (ab8227, 1:1000, Abcam) were added for overnight incubation on a shaker at 4°C. Following additional washes, HRP-conjugated goat anti-rabbit IgG secondary antibodies (ab288151, 1:2000, Abcam) were added for 1 hour incubation at room temperature. Using an ECL chromogenic substrate (P0018S, Beyotime, Shanghai, China), the films were further washed with TBST, and the corresponding bands were visualized with an imaging system. The relative density of the proteins was quantified using ImageJ software.

### 2.12 ELISA Assay

The levels of TNF-α, IL-6, and IL-1β (pro-inflammatory cytokines) were quantitatively assessed using the ELISA Kit (human TNF-α: PT518; IL-6: PI330; IL-1β: PI305; all from Beyotime). According to the provided protocol, cell culture supernatants were prepared and incubated with biotinylated detection antibodies specific for TNF-α, IL-6, and IL-1β supplied in the kits, followed by reacting with HRP-labeled streptavidin. Finally, the TMB chromogenic substrate was added to develop color. The absorbance values at 450 nm were read on a microplate reader. The levels of TNF-α, IL-6, and IL-1β were calculated based on the standard curves provided and expressed in picograms per milliliter (pg/mL) [25].

### 2.13 Cell Apoptosis Determination

Following the experimental treatment of THP-1-derived macrophages, approximately 2×10^5^ cells were collected and centrifuged. The cells were resuspended with Annexin V-FITC and PI solution from the apoptosis detection kit (C1062S, Beyotime, Shanghai, China) and incubated in the dark for 15 minutes. Subsequently, the cells were analyzed using a flow cytometer (Cytek Biosciences, Fremont, California, USA), and the apoptosis of each group was analyzed with FlowJo software [26].

### 2.14 Statistical Analysis

Statistical analyses were conducted in the R language (version 3.6.0). Two-group differences of continuous variables were evaluated by the Wilcoxon rank-sum test, while correlations were calculated by the Spearman method. Survival differences were analyzed with the log-rank test. All experiments were conducted in independent triplicate, and data were expressed in the form of mean ± SD. Data comparison was performed by one-way or two-way ANOVA or an unpaired t-test. A *p*-value<0.05 indicated statistical significance.

## 3 RESULTS

### 3.1 Differences in HDACs Among Sepsis Patients

**Fig. (1):**
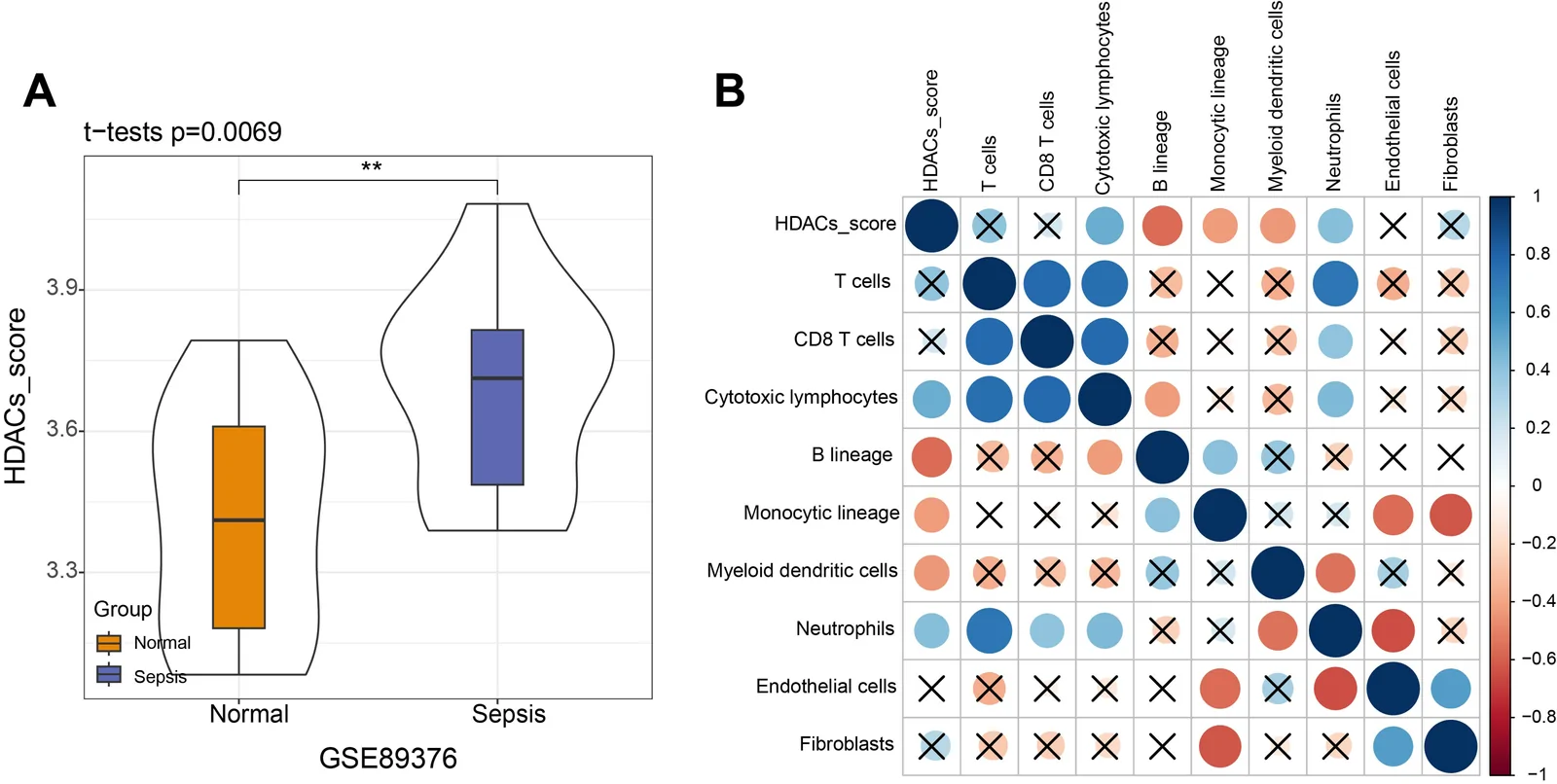
Differences in HDACs and correlation with immune cell scores in sepsis patients. (**A**) Comparison of HDACs scores by ssGSEA between Sepsis and Normal samples in the GSE89376 dataset; (**B**) Association between HDACs scores and the scores of 10 immune cell types calculated by the MCPcounter package. ** *p* <0.01.

The ssGSEA showed that the HDACs score in the Sepsis group was significantly higher than that in the Normal group, indicating a state of dysregulation of HDACs in the Sepsis group (Fig. **1A**). Subsequently, the correlation analysis between the HDACs score and scores of immune cells revealed a significant positive correlation between the HDACs score and cytotoxic lymphocytes and neutrophils, and a significant negative association with B lineage, monocytic lineage, and myeloid dendritic cells (Fig. **1B**).

### 3.2 Identification of HDAC-Related Gene Modules Based on WGCNA

**Fig. (2):**
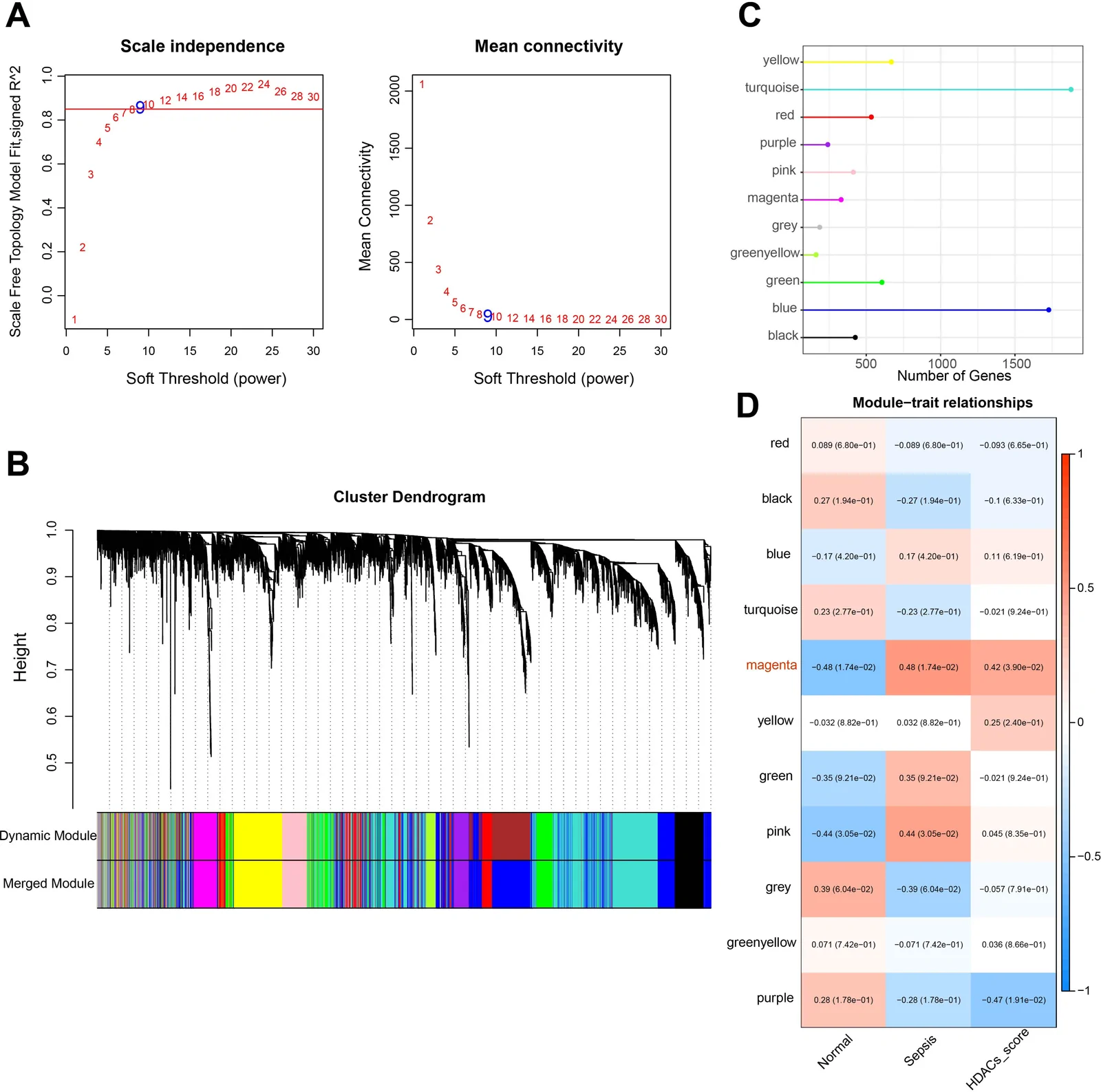
Expression of key module genes. (**A**) Various soft threshold powers (β) by scale-free fitting index analysis; various soft threshold powers assessed by mean connectivity analysis; (**B**) Dendrogram of genes clustered according to 1-TOM; (**C**) Number of genes in each module; (**D**) Association between traits for each module and module eigengenes.

We applied WGCNA to classify HDAC-associated gene modules. Samples were clustered to screen for co-expression modules. Sample clustering and a soft threshold of β=9 (Fig. **2A**) were selected for network classification. Subsequently, hierarchical clustering identified 11 co-expression modules after merging (Fig. **2B**), with the grey module comprising unassigned genes. The number of genes in each module is illustrated in Fig. (**2C**). Further analysis revealed that HDAC scores were strongly correlated with the magenta module (Fig. **2D**), significantly positively correlated with the sepsis group, and negatively correlated with the normal group.

### 3.3 Functional Enrichment Analysis Reveals Biological Mechanisms

**Fig. (3):**
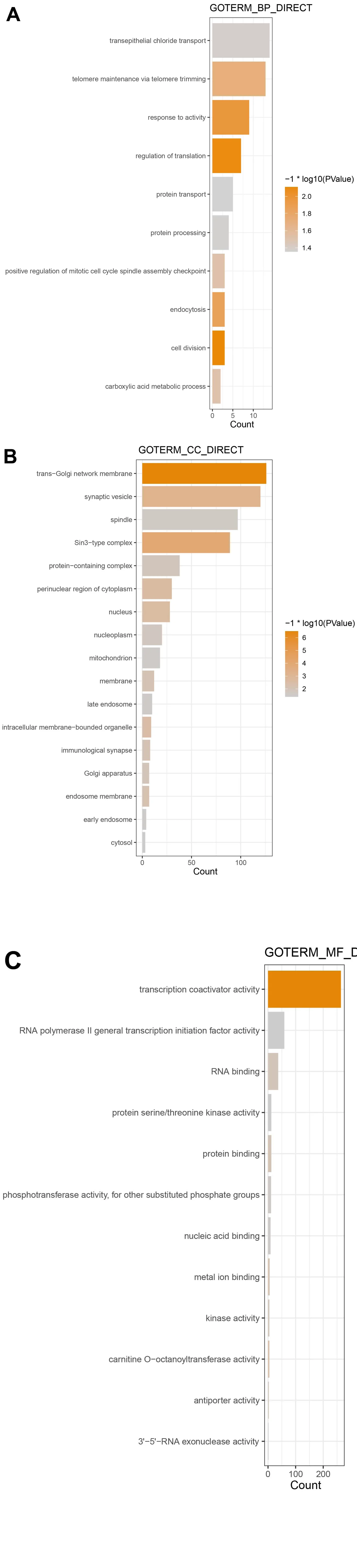
Enrichment analysis results of magenta module genes. (**A**) GO enrichment analysis in BP term; (**B**) GO enrichment analysis in CC term; (**C**) GO enrichment analysis in MF term.

Enrichment analysis was performed on the genes of the magenta module. Figs. (**3A**-**3C**) show the results of the enrichment analysis of the magenta module genes. In the Biological Process (BP) term, these genes were primarily enriched in pathways such as positive regulation of mitotic cell cycle spindle assembly checkpoint, cell division, protein processing, and carboxylic acid metabolic process (Fig. **3A**). In the Cellular Component (CC) term, these genes were mainly enriched in pathways like the trans-Golgi network membrane, synaptic vesicle, and Golgi apparatus (Fig. **3B**). In the Molecular Function (MF) term, these genes were predominantly enriched in pathways including transcription coactivator activity, RNA polymerase II general transcription initiation factor activity, and RNA binding (Fig. **3C**).

### 3.4 Machine Learning for Selecting Sepsis Biomarkers

**Fig. (4):**
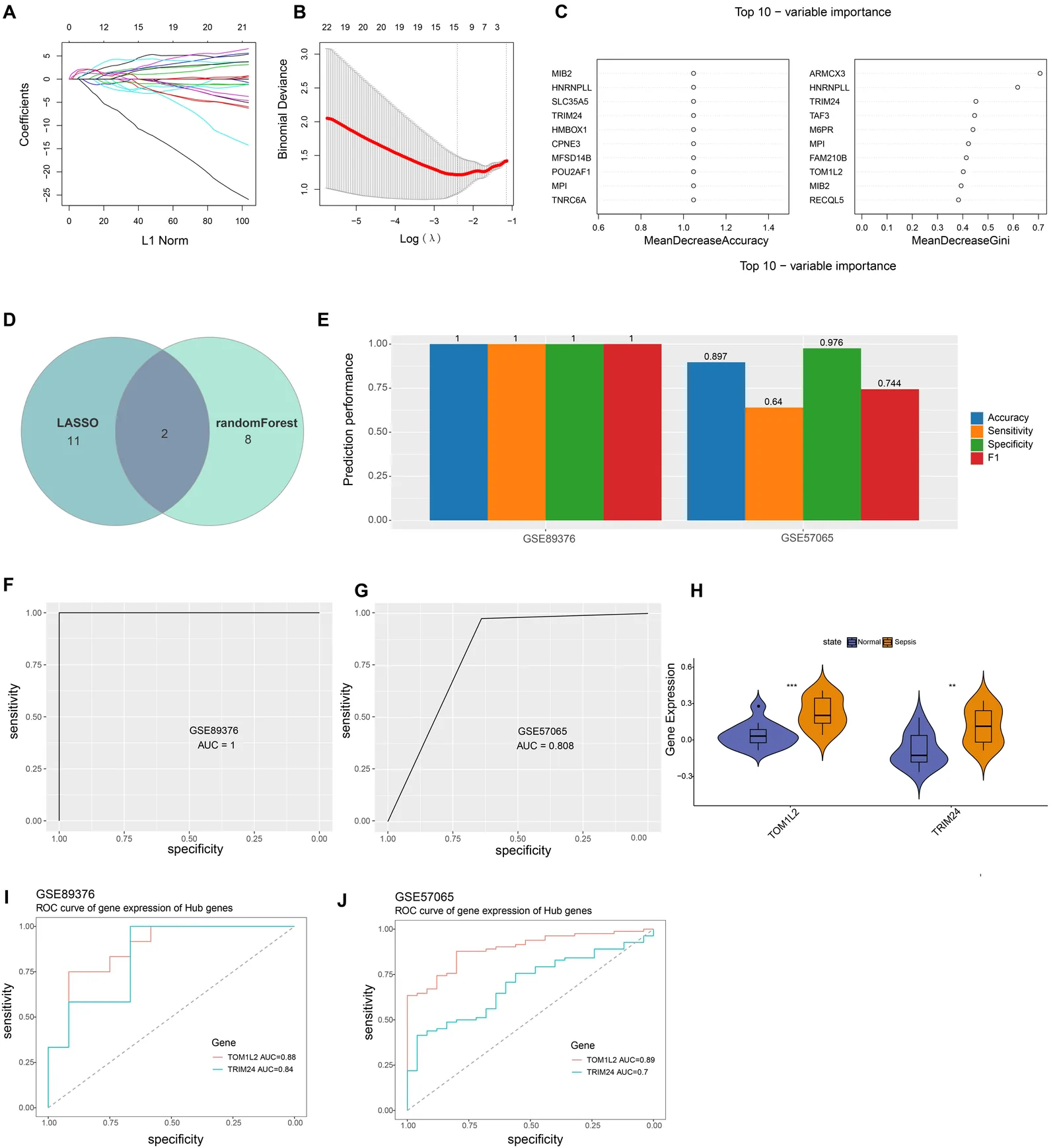
Machine learning for sepsis biomarker selection. (**A-B**) Changes in the coefficients of gene features and selection of the optimal penalty parameter (λ) in the LASSO regression model based on cross-validation; (**C**) Based on the random forest model, the importance of features was assessed using MeanDecreaseAccuracy and MeanDecreaseGini, and the top 10 contributing markers were displayed; (**D**) The intersection of feature genes screened by both random forest and LASSO analyses was shown in Venn diagram; (**E**) Performance metrics of the diagnostic model in each dataset; (**F**) ROC curve of the diagnostic model in GSE89376; (**G**) ROC curve of the model performance in GSE57065; (**H**) Violin plots showing the expression distribution of feature genes in the normal control group and the Sepsis group in GSE89376; (**I**) ROC curves and corresponding AUC value of feature genes in the GSE89376 dataset; (**J**) ROC curves and corresponding AUC value of the feature genes in the GSE57065 dataset. ** *p* <0.01, *** *p* <0.001.

LASSO regression analysis was employed to screen genes. Fig. (**4A**) illustrates the changes in regression coefficients of different gene features with the penalty parameter (λ) in LASSO regression. The optimal λ, selected *via* 10-fold cross-validation (Fig. **4B**), balanced model sparsity and predictive performance. Separately, random forest analysis ranked the top 10 markers by MeanDecreaseAccuracy and MeanDecreaseGini (Fig. **4C**). A Venn diagram (Fig. **4D**) shows that LASSO selected 11 genes, random forest selected 8 genes, with two common genes (*TOM1L2* and *TRIM24*). In the GSE89376 and GSE57065 datasets, the Specificity was higher than 0.95, and the Accuracy was higher than 0.8 (Fig. **4E**). These two hub genes were validated as highly expressed in sepsis within the GSE89376 dataset (Fig. **4H**). Our diagnostic model, built on these features, showed Specificity >0.95 and Accuracy >0.8 in external datasets (GSE89376, GSE57065; Fig. **4E**), with AUC >0.8 indicating strong performance (Figs. **4F** and **G**). Individually, each hub gene achieved an AUC >0.7 in both datasets (Figs. **4I** and **J**).

### 3.5 Correlation Between Hub Genes and Immunity

**Fig. (5):**
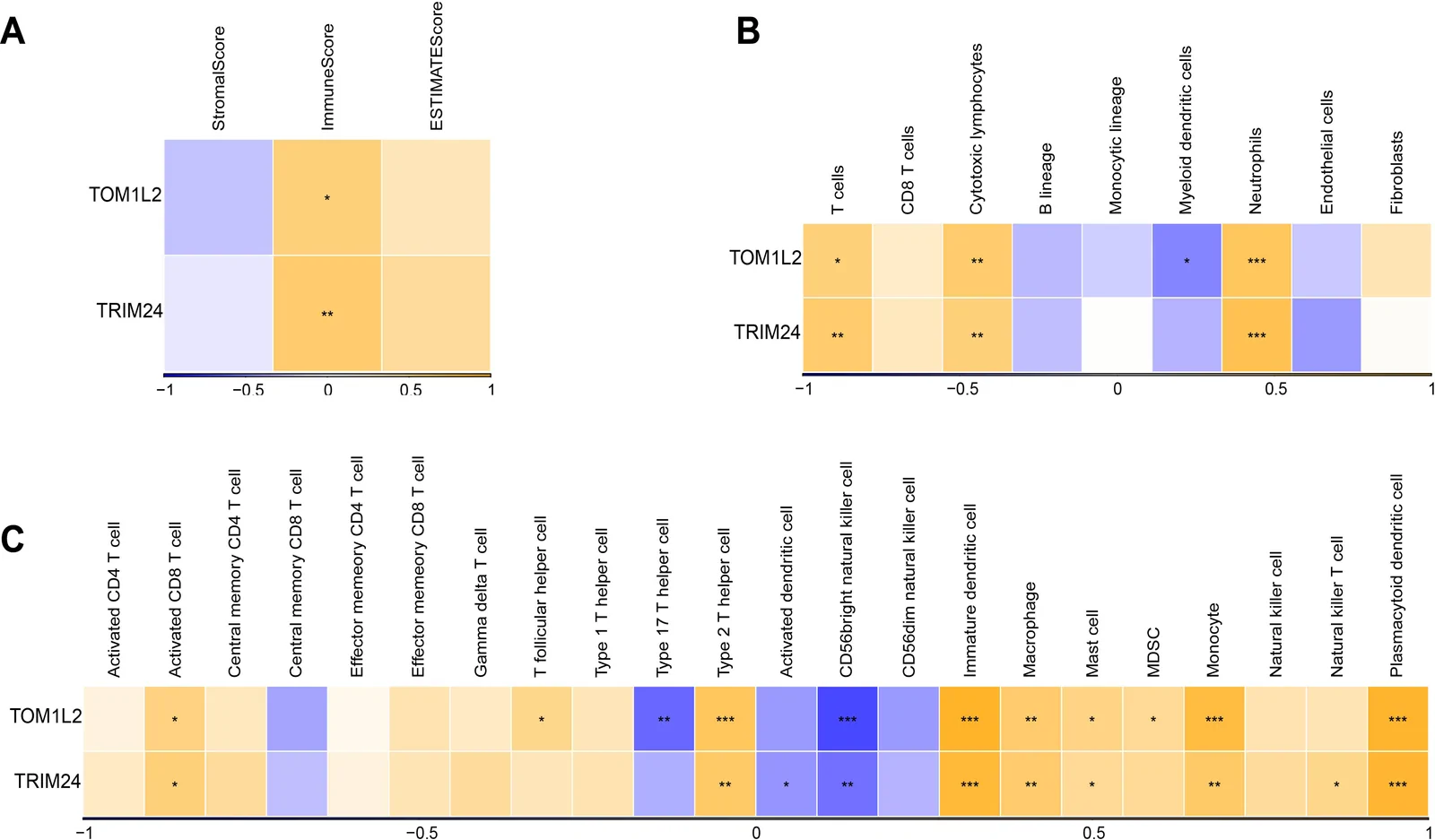
Correlation analysis of immune microenvironment and gene expression. (**A**) Correlation between ESTIMATE scores and the two hub genes; (**B**) Correlation between MCP-counter scores and the two hub genes; (**C**) Correlation between ssGSEA scores and the two hub genes. Yellow and purple indicate positive and negative correlations, respectively. *** *p* <0.001, ** *p* <0.01, * *p* <0.05.

The ESTIMATE algorithm showed that the two hub genes (*TOM1L2* and *TRIM24*) were significantly positively correlated with ImmuneScore in the GSE89376 dataset (Fig. **5A**). The MCP-counter (Fig. **5B**) showed that these two hub genes were significantly positively correlated with T cells, cytotoxic lymphocytes, and neutrophils. The ssGSEA function of GSVA also revealed that *TOM1L2* and *TRIM24* were significantly positively correlated with the scores of activated CD8 T cells, type 2 T helper cells, mast cells, monocytes, plasmacytoid dendritic cells, immature dendritic cells, and macrophages (Fig. **5C**).

### 3.6 Drug Prediction for Hub Genes and Molecular Docking

**Fig. (6):**
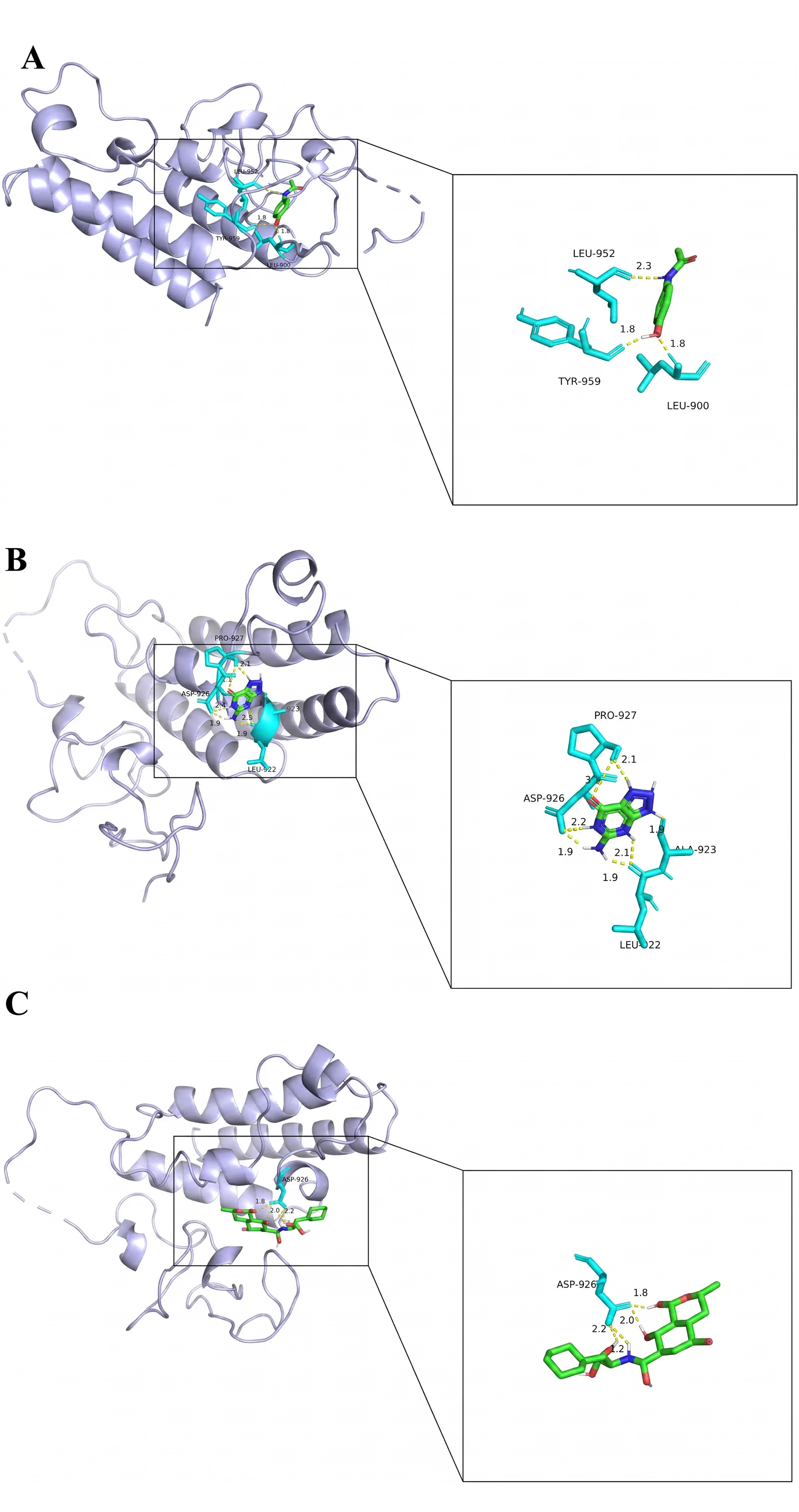
Results of molecular docking. (**A**) The results of molecular docking for *TRIM24* and acetaminophen; (**B**) The results of molecular docking for *TRIM24* and 8-azaguanine; (**C**) The results of molecular docking for *TRIM24* and ochratoxin A. In the figures, the blue molecule represents the receptor protein, the green molecule indicates the drug small molecule, and the cyan represents amino acids. Hydrogen bonds between the receptor protein and the drug small molecule are indicated by yellow dashed lines, with the numbers above representing the hydrogen bond lengths in units of angstroms (Å).

*TOM1L2* and *TRIM24* were input into the Enrichr package to predict drugs targeting these biomarkers in the DSigDB database. After screening, *TRIM24* was selected as the protein for molecular docking because *TOM1L2* only had a predicted 3D structure. Using the crystal structure of TRIM24 (PDB: 4YBM), we docked three candidate drugs (acetaminophen, 8-azaguanine, and ochratoxin A). The docking results showed that the binding energies between *TRIM24* and the three drug molecules were -5.83 kcal/mol for Acetaminophen, -6.59 kcal/mol for 8-azaguanine, and -6.07 kcal/mol for Ochratoxin A, with all hydrogen bond lengths within the normal range (Figs. **6A**-**C**). The results and parameters of the molecular docking are presented in Tables **2** and **3**.

### 3.7 Candidate Gene Validation and Functional Dissection in a Cellular Sepsis Model

**Fig. (7):**
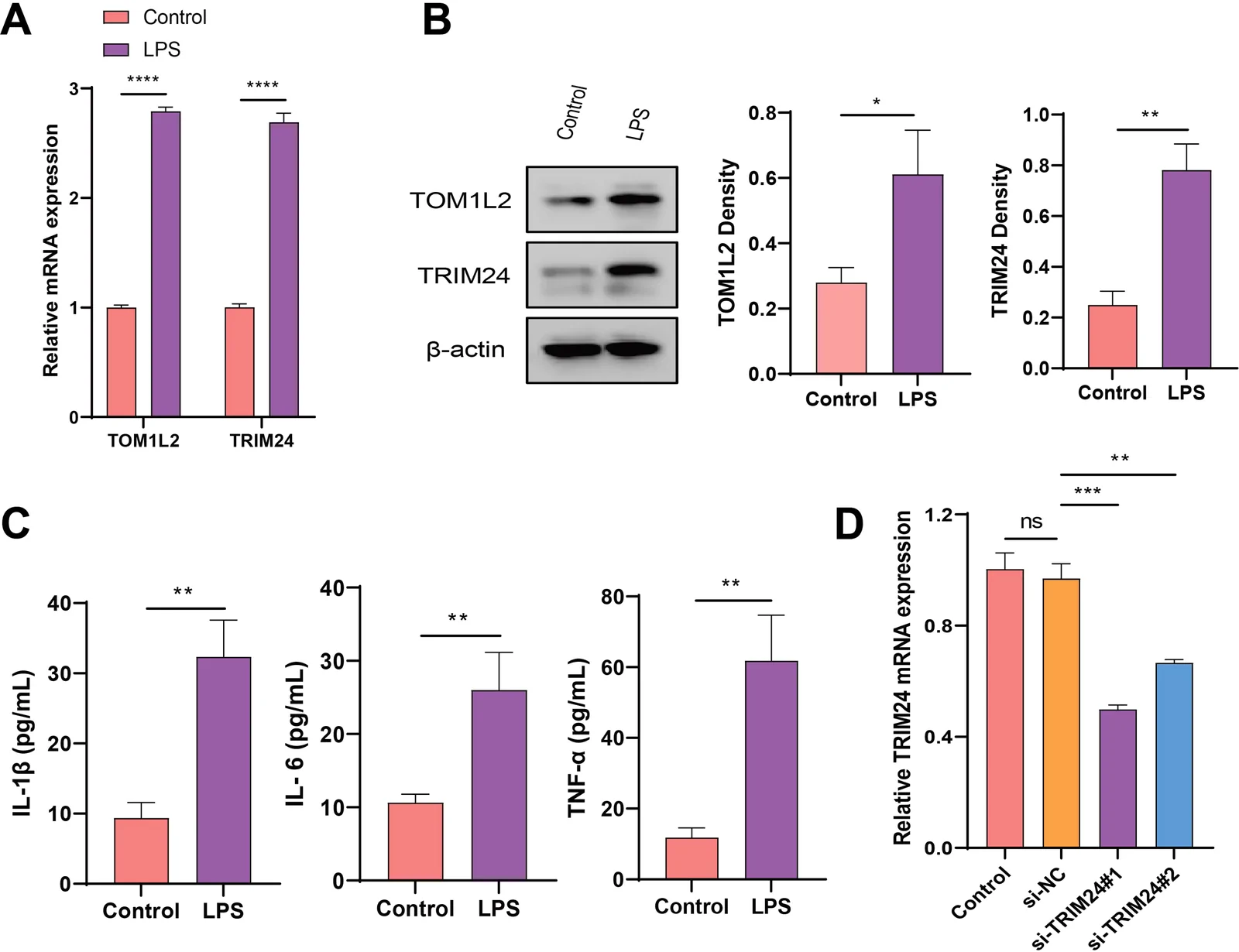
Effects of LPS-Induced Inflammation and the Impact of *TRIM24* Knockdown on Inflammatory Cytokines. (**A**)The mRNA and (**B**) protein expressions of *TOM1L2* and *TRIM24*; (**C**) Quantitative analysis of inflammatory factor levels *via* ELISA; (**D**) The effect of *TRIM24* knockdown on the expression of *TRIM24*. Data were expressed as SD ± mean, ****p <0.0001, ***p <0.001; **p <0.01; *p <0.05.

**Fig. (8):**
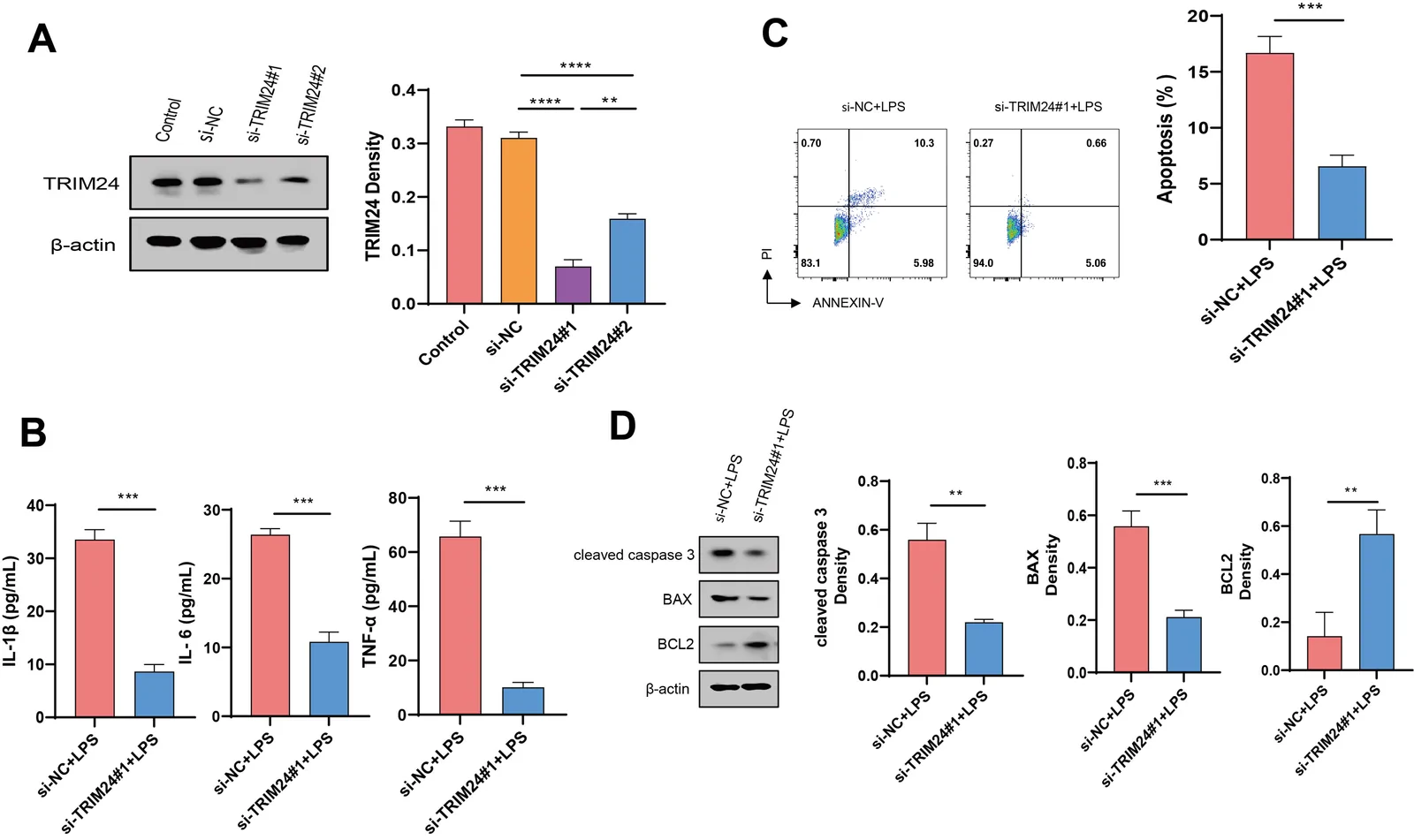
Impact of *TRIM24* knockdown on LPS-stimulated inflammatory response and apoptosis in THP-1-derived macrophages. (**A**) Protein expression and density of *TRIM24*; (**B**) Quantitative analysis of inflammatory cytokine (TNF-α, IL-1β, IL-6) secretion levels detected by ELISA; (**C**) Flow cytometry assessment of apoptosis levels; (**D**) Expression detection of apoptosis-correlated proteins (BCL2, cleaved caspase 3, BAX). Data were expressed as mean ± SD, ****p <0.0001; ***p <0.001; **p <0.01.

The qPCR results demonstrated that the mRNA levels of both genes in the LPS-treated group were significantly higher (Fig. **7A**), thereby validating the upregulation of *TOM1L2* and *TRIM24* by LPS at the transcriptional level. Furthermore, compared with the Control group, the findings revealed that the relative density values of *TOM1L2* and *TRIM24* proteins in the LPS group were notably higher (Fig. **7B**). The ELISA data also indicated that the concentrations of all three pro-inflammatory factors in the LPS group were significantly higher than those in the Control group (Fig. **7C**), confirming that LPS successfully induced a sepsis-like inflammatory response in THP-1-derived macrophages. Given the established role of *TRIM24* in immune regulation and its broader correlation with the septic immune microenvironment, it was selected for subsequent in-depth functional validation. Subsequently, we validated the knockout efficiency of *TRIM24* (Figs. **7D** and **8A**). As shown in Fig. (**8B**), the si-*TRIM24*#1+LPS group exhibited notably lower levels of all three pro-inflammatory cytokines than the control group, indicating that knockdown of *TRIM24* can ameliorate the inflammatory response in the sepsis model. Flow cytometry data revealed that cell apoptosis in the si-*TRIM24*#1+LPS group was notably lower than that in the si-NC+LPS group, directly validating that knockdown of *TRIM24* inhibited apoptosis in THP-1-derived macrophages in the sepsis model *in vitro* (Fig. **8C**). Following knockdown of *TRIM24* in the *in vitro* sepsis model, the levels of pro-apoptotic proteins, cleaved caspase 3, and BAX were reduced, while the level of the anti-apoptotic protein BCL2 was increased. This suggested that knockdown of *TRIM24* inhibited apoptosis in THP-1-derived macrophages in the sepsis model (Fig. **8D**).

## 4 DISCUSSION

The diagnosis and treatment of sepsis remain challenging [27, 28]. At present, the role of epigenetics in the pathogenesis of sepsis has gradually attracted research attention [29]. Histone acetylation, a key epigenetic modification, is implicated in a variety of biological processes, including immune regulation, inflammatory response, and cellular metabolism [30]. It has been demonstrated that histone acetylation regulates inflammation and immune cells in sepsis, thereby influencing disease progression. Additionally, the associated gene network offers new directions for sepsis detection [31].

We screened two relevant feature genes (*TOM1L2* and *TRIM24*) through machine learning algorithms. *TOM1L2*, a member of the TOM1 family, is a membrane transport protein containing VHS, GAT, and clathrin-binding domains, which mediate vesicular transport and endosomal sorting [32]. *TOM1L2* has also been identified as a potential causal target for Alzheimer's Disease (AD) [33] and pancreatic cancer [34], but its role in sepsis is less studied. Given its function in intracellular vesicular transport and signal transduction, particularly of K63-ubiquitinated proteins, it is speculated that *TOM1L2* may influence the inflammatory response and cellular dysfunction in sepsis [32]. However, further exploration of its specific mechanisms of action in sepsis is still needed in the future.

*TRIM24* is a multifaceted protein containing a tripartite motif with emerging significance in epigenetic and inflammatory regulation [35, 36]. Notably, its function as a “histone reader” places it within the core of epigenetic modification, suggesting potential functional interplay or shared regulatory pathways with HDACs. In the context of inflammation and sepsis, *TRIM24* acts as an inflammatory negative regulator in macrophages by inhibiting the IFNβ/IL-10 axis to fine-tune cytokine production, a process where dynamic histone acetylation is known to be pivotal [37, 38]. This regulatory role implied that *TRIM24*-mediated transcriptional outcomes may intersect with HDAC activity, which governs the accessibility of inflammatory gene loci. Furthermore, its capacity as an E3 ubiquitin ligase opens the possibility for post-translational regulation of HDACs or their co-factors *via* ubiquitination, potentially influencing the stability or assembly of epigenetic complexes involved in sepsis-related cellular stress [36]. Beyond immunity, in cancers such as Metaplastic Breast Cancer (MpBC), *TRIM24* drives oncogenic signaling by binding directly to gene promoters and activating pathways like PI3K/AKT/mTOR [39]. This transcriptional control mechanism further underscores its role within the epigenetic landscape, where HDACs often serve as complementary or antagonistic regulators of gene expression. Collectively, these functions position *TRIM24* not only as a key inflammatory and oncogenic modulator but also as a plausible functional connector or downstream effector within HDAC-influenced regulatory networks, demanding future investigation into its direct crosstalk with HDAC-mediated deacetylation in sepsis pathogenesis.

Acetaminophen, 8-azaguanine, and Ochratoxin A were drug molecules for docking. Recent studies have linked the early use of Acetaminophen to reduced mortality and shorter ICU stays in both sepsis [40, 41] and related ARDS [42]. 8-Azaguanine is a triazole analog of guanine, which was initially discovered in a variant of Streptomyces albus, and possesses potent antimicrobial and antitumor activities [43]. At present, the role of 8-Azaguanine in sepsis has been rarely investigated. Ochratoxin A is a mycotoxin produced by several types of fungi and is known for its diverse toxic effects, including immunotoxicity [44, 45]. It has been found that Ochratoxin A can induce immunotoxicity in zebrafish by targeting Annexin A1-mediated neutrophil apoptosis [46], suggesting that Ochratoxin A may negatively impact the immune system by affecting the function of immune cells. Thus, the immunotoxicity of ochratoxin A may be associated with immune suppression in sepsis.

Sepsis could impair both the innate and adaptive immune responses of the host, subsequently increasing the risk of primary and secondary infections [47]. Various types of immune cells are involved in this process, such as neutrophils, lymphocytes, and monocytes/macrophages, which recognize and phagocytose pathogens and trigger the release of cytokines, thereby activating other cells [48]. Our study revealed significant differences in the scores of immune cells, such as activated CD8 T cells, type 2 T helper cells, mast cells, monocytes, immature dendritic cells, macrophages, T cells, cytotoxic lymphocytes, and neutrophils in relation to *TOM1L2* and *TRIM24*. Macrophages, which possess immunosuppressive functions, exert detrimental effects in sepsis through *CASP11*-dependent pyroptosis [48, 49]. It has been confirmed that immune cells play a decisive role in the pathogenesis of sepsis [50].

Despite the promising findings, this study has several limitations that should be acknowledged. Firstly, the computational analysis was predominantly based on public transcriptomic datasets. While the *in vitro* validation strengthens the conclusions, the diagnostic and prognostic value of the identified biomarkers (*TOM1L2* and *TRIM24*) requires further confirmation in larger, multi-center prospective clinical cohorts with diverse patient demographics and stratified sepsis etiologies. Future studies should collect matched blood samples at different stages of sepsis to dynamically assess their expression and correlation with clinical severity scores and outcomes. Secondly, the candidate biomarkers were screened from modules correlated with the HDAC activity signature, yet this study did not experimentally elucidate whether they were direct transcriptional targets of specific HDACs. Future work should employ chromatin immunoprecipitation sequencing for relevant HDACs and treat primary immune cells or cell lines with specific HDAC inhibitors/agonists to examine the causal regulatory relationship between HDAC activity and the expression of these genes. Thirdly, while molecular docking predicted potential interactions between *TRIM24* and compounds like acetaminophen, these were computational simulations. The actual binding affinity, functional consequences of binding, and therapeutic efficacy need validation through *in vitro* binding assays, functional cellular assays, and ultimately, preclinical studies in animal models of sepsis. Fourthly, the functional validation was strategically focused on *TRIM24* due to its stronger mechanistic links to inflammation. The role of *TOM1L2* in sepsis pathogenesis remained unexplored and required independent investigation using knockdown/overexpression approaches followed by comprehensive phenotyping. Finally, the immune infiltration analysis, while comprehensive, established correlations rather than causal mechanisms. To understand how these biomarkers might shape the immune microenvironment, future research should employ techniques such as scRNA-seq of patient samples, co-culture systems of genetically modified macrophages with other immune cells, and analysis of cytokine secretion profiles to dissect their specific impact on immune cell differentiation, recruitment, and function. Addressing these limitations in subsequent research will be crucial for translating these biomarker discoveries into clinically applicable diagnostic tools and targeted therapeutic strategies.

## CONCLUSION

In conclusion, this study leveraged the dysregulated HDAC activity signature as a screening framework to identify novel biomarkers for sepsis. Through integrated computational analyses, we pinpointed *TOM1L2* and *TRIM24* as robust diagnostic biomarkers closely associated with immune microenvironment alterations in sepsis. The *in vitro* validation confirmed that LPS significantly upregulated their expression in macrophages, and established that knockdown of *TRIM24* attenuated the inflammatory response and inhibited cellular apoptosis. These findings provided a valuable two-gene model for the potential early detection of sepsis and highlighted *TRIM24* as a promising functional target for modulating dysregulated immune responses in this condition.

## Figures and Tables

**Table 1:** specific primer sequence for qPCR.

Gene	Forward (5’-3’)	Reverse (5’-3’)
TOM1L2	TGTCGGAGGACTTGCTTCTGCA	CCACACCTCAATGTCGTCCATG
TRIM24	CCAGAACATACCACGACAAGCAA	GGAGTAGAGGATGTGCTGTTGG
GAPDH	GTCTCCTCTGACTTCAACAGCG	ACCACCCTGTTGCTGTAGCCAA

**Table 2:** Molecular docking binding energy.

Compound CID	Molecula_name	Gene_name	PDB_ID	Energy (kcal/mol)
1983	acetaminophen	TRIM24	4YBM	-5.83
135403646	8-azaguanine	TRIM24	4YBM	-6.59
442530	ochratoxin A	TRIM24	4YBM	-6.07

**Table 3:** Molecular docking parameters.

Term	Spacing	Npts	Center
TRIM24_acetaminophen	0.353	126 110 126	6.144, -13.486, -16.879
TRIM24_8-azaguanine	0.375	126 110 126	7.458, -13.178, -18.346
TRIM24_ochratoxin A	0.375	126 124 122	7.733, -14.764, -16.974

## Data Availability

The datasets generated and/or analyzed during the current study are available in the (GSE57065) repository, (https://www.ncbi.nlm.nih.gov/geo/query/acc.cgi?acc=GSE57065), and (GSE89376) repository, (https://www.ncbi.nlm.nih.gov/geo/query/acc.cgi?acc=GSE89376).

## LIST OF ABBREVIATIONS

AD: Alzheimer's Disease
AUC: Area Under the Curve
ARDS: Acute Respiratory Distress Syndrome
ATCC: American Type Culture Collection
BP: Biological Processes
CC: Cellular Components
CFH: Cell-free Hemoglobin
EMT: Epithelial-mesenchymal Transition
ELISA: Enzyme-linked Immunosorbent Assay
FBS: Fetal Bovine Serum
GO: Gene Ontology
GEO: Gene Expression Omnibus
HATs: Histone Acetyltransferases
HDACs: Histone Deacetylases
HRP: Horseradish Peroxidase
IL-10: Interleukin-10
LASSO: Least Absolute Shrinkage and Selection Operator
LPS: Lipopolysaccharide
miRNA: Microrna
MpBC: Metaplastic Breast Cancer
MF: Molecular Functions
PMA: Phorbol 12-myristate 13-acetate
PI: Propidium Iodide
ROC: Receiver Operating Characteristic
qPCR: Quantitative PCR
qRT-PCR: Quantitative Reverse Transcription Polymerase Chain Reaction
ssGSEA: Single-sample Gene Set Enrichment Analysis
SAE: Sepsis-associated Encephalopathy
siRNA: Small Interfering RNA
si-NC: Small Interfering Negative Control
WGCNA: Weighted Gene Co-expression Network Analysis
TOM: Topological Overlap Matrix
TNF-α: Tumor Necrosis Factor-alpha
TIICs: Tumor-infiltrating Immune Cells
TNBC: Triple-negative Breast Cancer
OOB: Out-of-bag
IL-6: Interleukin-6
IL-1β: Interleukin-1β
